# Attitudes of undergraduate university women towards HPV vaccination: a cross-sectional study in Ottawa, Canada

**DOI:** 10.1186/s12905-018-0622-0

**Published:** 2018-08-02

**Authors:** Rachel Fernandes, Beth K. Potter, Julian Little

**Affiliations:** 1 School of Epidemiology and Public Health, University of Ottawa, Ottawa, Canada; 2 Faculty of Medicine, School of Epidemiology and Public Health, University of Ottawa, 600 Peter Morand Crescent, Room 207G, Ottawa, On K1G 5Z3 Canada; 3 Children’s Hospital of Eastern Ontario Research Institute, Ottawa, Canada; 4 Canada Research Chair in Human Epidemiology, Ottawa, Canada

**Keywords:** HPV, Vaccination, Sexual health, Sexual behaviour

## Abstract

**Background:**

Persistent infection with certain subtypes of human papillomavirus (HPV) is a necessary cause of cervical cancer. Although two prophylactic vaccines have been licensed in Canada against cancerous subtypes of HPV, vaccine uptake has been lower than anticipated. The primary objective of this study was to determine the acceptability of catch-up HPV vaccination to undergraduate university women under the age of 25, by assessing their perceptions of HPV vaccination.

**Methods:**

A total of 401 University of Ottawa female undergraduate students participated in a cross-sectional bilingual web-based survey on HPV vaccination.

**Results:**

The prevalence of immunization with at least 1 HPV vaccine dose was 49% in the study population. Although the overall attitude of study participants towards the vaccine was positive, vaccinated respondents had a more favourable attitude towards the vaccine than non-vaccinated respondents. Approximately half of the non-vaccinated respondents were interested in receiving the vaccine at some point in the future. The primary barriers to HPV vaccination identified by non-vaccinated respondents were lack of knowledge about the vaccines, potential vaccine side effects and cost of vaccination. Multivariable analysis comparing non-vaccinated respondents who intended to be vaccinated and those who did not suggests that the former group had a more favourable attitude towards the vaccine and would be influenced by doctor recommendation.

**Conclusions:**

Offering HPV vaccination for women aged 18 to 25 provides an opportunity to address suboptimal vaccination coverage in the population and may reduce health inequities demonstrated by variations in cervical cancer incidence within jurisdictions.

**Electronic supplementary material:**

The online version of this article (10.1186/s12905-018-0622-0) contains supplementary material, which is available to authorized users.

## Background

Cervical cancer is the second most common cancer afflicting women and the third leading cause of cancer mortality among women worldwide [1, 2]. This disease affects more than 530,000 women annually, 85% of whom live in the developing world [2]. Persistent infection with Human papillomavirus (HPV) has been established as the primary cause of cervical cancer, accounting for upwards of 90% of all cervical cancer cases [3]. The vast majority of HPV infections are asymptomatic or sub-clinical, which has contributed to the rapid transmission and spread of the virus [4, 5].

HPV infections are so commonly transmitted that nearly 75% of Canadian adults are infected with the virus at some point in their life [6] while the highest rates of infection are in the population under the age of 24 [6].

Three different HPV vaccines have been approved for use in Canada. Cervarix has been approved for females only, while Gardasil has been approved for both sexes. Both vaccines protect against high-risk HPV types 16 and 18, which together account for 70% of invasive cervical cancer cases [7]. Gardasil also provides immune protection to types 6 and 11, which cause genital warts. Although a nine-valent vaccine, Gardasil 9, is now available [8], only the quadrivalent Gardasil vaccine was in use at the time of the study. Though initially approved as a three-dose schedule, the new recommendation for immunocompetent girls aged 9–14 for both vaccines is a two-dose schedule, which was approved in July 2014 [9]. The potential advantages of HPV vaccination include prevention of HPV infection, cervical cancer and in the case of Gardasil, genital warts [10, 11]. Although these vaccines are most effective prior to onset of sexual activity, they can still be given to women who are sexually active, since it is rare for women to be infected with all vaccine-covered viral strains [12]. Furthermore, Cervarix offers cross-protection to additional oncogenic strains HPV-31, 33 and 45, while Gardasil may also offer cross-protection against strain 31 [3, 13].

Health Canada licensed the use of Gardasil in 2006 [14] and allocated $300 million to provinces and territories in 2007 to promote HPV vaccination in young girls [15]. The bivalent vaccine, Cervarix, was licensed for use in girls and women aged 10 to 25 in 2010 [16] (though now the recommendation is females aged 9 through 26), while the quadrivalent vaccine Gardasil has been approved for females aged 9 to 45 [12].

The province of Ontario offered HPV vaccination to girls in grade 8 through school programs at no cost to girls and their families. Despite an expectation from the Canadian government that vaccine coverage would be 80% for eligible girls in the first 2 years of vaccination, by the end of 2009 in Ontario, only 56.6% of eligible girls had received the first dose of the three dose vaccine series [15]. Of this proportion, 85.3% had received all three doses of the vaccine, resulting in an overall vaccine uptake of 48.3% in Ontario [15]. This uptake rate is insufficient to ensure herd immunity, which requires coverage of 80% of the population [17].

In addition to low vaccine uptake rates through Ontario school programs, many women did not meet the age criteria to benefit from free vaccination when it was initially recommended in 2007, as only girls in the eighth grade were initially targeted [18]. Thus, a sizeable number of Ontario women currently over the age of 18 years may benefit from being offered a HPV vaccine. To inform the current and any future catch-up HPV vaccination programs for young adult women in Ontario, it is therefore important to study women in this age group to assess their willingness to obtain the vaccine and factors associated with potential uptake.

The goal of this study was to determine the perceptions of women in university in Ottawa, aged 18 to 25, about the HPV vaccines. University women were chosen as the study population since as a well-educated subset of the population, any problems incurred with accepting or understanding the vaccine could be indicative of broader problems. Further, this population is diverse because Ottawa is a cosmopolitan city with substantial recent immigration from multiple countries [19]. Specific objectives were to:

1. Estimate the proportion of women in this age group who have been vaccinated with a HPV vaccine;
2. Estimate the proportion of women who would be interested in receiving the vaccine, among those who have not been vaccinated; and
3. Determine the main barriers that prevent and factors that promote HPV vaccination.

## Methods

We conducted a cross-sectional study by means of a web-based survey. Undergraduate women between the ages of 18 and 25 who attended the University of Ottawa during the winter semester of 2013 were eligible for the study. We identified administrative, faculty, and student association contacts for a range of faculties and departments across the university and approached them to ask whether they would agree to administer our survey. Those who agreed were responsible for directly contacting their students using already compiled electronic lists that were inclusive of all students in a given faculty or department.

Students from one faculty and one department were contacted three times while the remaining potential participants were contacted once via email. These emails contained links to the web-based survey and were distributed in both English and French, as the University is bilingual.

The instrument, a study-specific bilingual questionnaire available on the Fluid Surveys web platform, was developed using previously published instruments [20, 21] and original questions (See Additional file 1 and Additional file 2). The questionnaire followed one of two automated branches, depending on the vaccination status of the participant. It had a total of 35 questions. The questionnaire assessed attitude towards receiving the HPV vaccine, beliefs about HPV vaccination, intentions to receive or complete the vaccine series, barriers to vaccination, factors that promote vaccine uptake and knowledge. The Theory of Planned Behaviour (TPB) [22] was used to design questions for the sections of the questionnaire pertaining to attitudes, beliefs and intentions toward HPV vaccination. The TPB has previously been used to predict health related behaviours in university-aged populations [21, 23]. According to the theory, intention is the best predictor of behaviour. Intention itself is predicted by attitude toward a specific behaviour, subjective or social norms about the behaviour and one’s perception of their own control over the specific behaviour [22]. Subjective norms represent perceived social pressure to participate in the behaviour. The questionnaire assessed these concepts through a series of seven-point Likert scales, to assign a quantitative value to each construct. As a measure of overall attitude toward vaccination, the mean score of a series of four behavioural belief variables (defined as a person’s belief about the consequences of specific behaviour, particularly HPV vaccination) was taken. A lower score represented a more positive attitude toward the vaccine series, while a higher score indicated an unfavourable attitude. The primary outcomes of the study were the proportion of participants who were vaccinated against HPV and the intention of non-vaccinated participants to receive the vaccine series. Being vaccinated against HPV was defined as having received a minimum of one vaccine dose of either HPV vaccine.

The data from the questionnaire were analyzed using descriptive statistics, including means and standard deviations for continuous variables and proportions with their associated 95% confidence intervals for categorical variables. Subgroup analysis using univariable linear regression was used to compare the respondents who intended to be vaccinated against HPV at some point in the future with those who did not intend to be vaccinated in the future. Multivariable logistic regression was conducted to identify predictors of intended acceptance of HPV vaccination by assessing the differences between these two subpopulations. Variables representing the three pillars of the TPB, attitude, subjective norms and perceived behavioural control were included as predictors in the regression model, in addition to other participant characteristics which were significant at a 0.05 level when modelled individually against the outcome. Demographic characteristics previously associated with HPV vaccination in the literature were included in the final logistic regression model in order to control for possible confounding [21]. The database is available as an Additional file (see Additional file 3).

We handled item-missing data by casewise deletion, excluding participants who did not complete the first section of the questionnaire, including HPV vaccination status, attitudes towards the vaccine and intentions towards future vaccination. Statistical analyses were conducted using SAS 9.3.

This study was approved by the Ottawa Hospital Research Ethics Board (20130038-01H). Written informed consent was completed online prior to beginning the survey.

## Results

### Participants

Of the 2398 undergraduate women from four faculties (Arts, Social Sciences, Science and Health Sciences) who were invited to participate in this study, 378 completed the full questionnaire, while 23 others completed at least its first section. Approximately 15% of these respondents completed the survey in French. The study’s response rate was estimated to be 17%.

### Descriptive data

Table 1 describes the general demographics of the study participants. The average age of study participants was 20.4, while the median ages for the vaccinated and non-vaccinated cohorts were 20 and 21 years, respectively. This study included women who had lived in 8 different Canadian provinces and several countries, and includes several ethnicities.

**Table 1:** Participant characteristics by vaccination status

Characteristic	Vaccinated (*n* = 196)		Not vaccinated (*n* = 205)	
	*N*	%	*N*	%
Age				
≤ 19	77	39.29	44	21.46
20–21	73	37.24	93	45.37
22–23	34	17.35	36	17.56
≥ 24	3	1.53	18	8.78
*Missing*	9	4.59	14	6.83
Faculty				
Arts	9	4.59	10	4.88
Health Sciences	17	8.67	13	6.34
Science	145	73.98	147	71.71
Social Sciences	16	8.16	20	9.76
*Missing*	9	4.59	15	7.32
Ethnicity				
White	144	73.47	115	56.10
Chinese	6	3.06	12	5.85
Black	8	4.08	15	7.32
Other	28	14.29	46	22.44
*Missing*	10	5.10	17	8.29
Province or Country of Residence before University of Ottawa				
Ontario	152	77.55	156	76.10
Quebec	22	11.22	15	7.32
Other Canadian Provinces	7	3.57	12	5.85
Outside Canada	6	3.06	7	3.41
*Missing*	9	4.59	15	7.32
Canadian Born				
Yes	164	83.67	154	75.12
No	24	12.24	35	17.07
*Missing*	8	4.08	16	7.80
Ever had Sex				
Yes	140	71.43	124	60.49
No	48	24.49	63	30.73
*Missing*	8	4.08	18	8.78
Pap Smear				
More than 1	68	34.69	57	27.80
Once	35	17.86	25	12.20
None	81	41.33	103	50.24
Don’t know	3	1.53	4	1.95
*Missing*	9	4.59	16	7.80
Abnormal Pap Smear				
Yes	14	7.14	18	8.78
No	159	81.12	155	75.61
Don’t know	13	6.63	15	7.32
*Missing*	10	5.10	17	8.29
Birth Control/Contraception				
Yes	128	65.31	95	46.34
No	59	30.10	93	45.37
*Missing*	9	4.59	17	8.29
HPV infection				
Yes	7	3.57	10	4.88
No	181	92.35	178	86.83
*Missing*	8	4.08	17	8.29

Of the 401 study participants, 196 (48.88%) had received at least one dose of the HPV vaccine series. Of this subset, 57.65% (113) had received all three doses, while 13 (6.63%) and 31 (15.81%) respondents had received one and two doses of the vaccine respectively; nearly 20% (38) of the respondents were unsure of the number doses they had received.

### Intentions toward vaccination

**Fig. 1:**
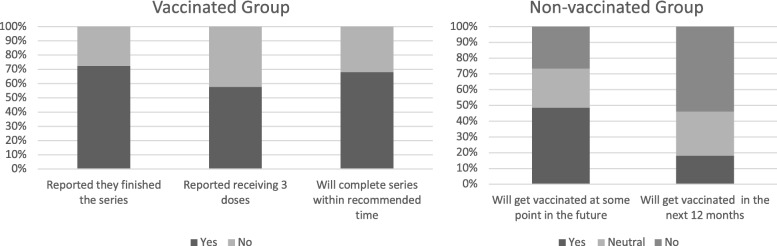
Vaccination intentions of vaccinated and unvaccinated groups

From the vaccinated cohort, 142 women (73.2%) specified that they had already received the whole series, although only 113 indicated they had received 3 vaccine doses (Fig. 1). Only 34 of the women who had not received all 3 doses intended to complete the vaccine series in the time frame scheduled. Two women indicated they would not be completing the series. Nearly 50% (*n* = 99) of the women in the unvaccinated group reported that they intended to be vaccinated at some point in the future.

### Attitudes towards HPV vaccination

**Fig. 2:**
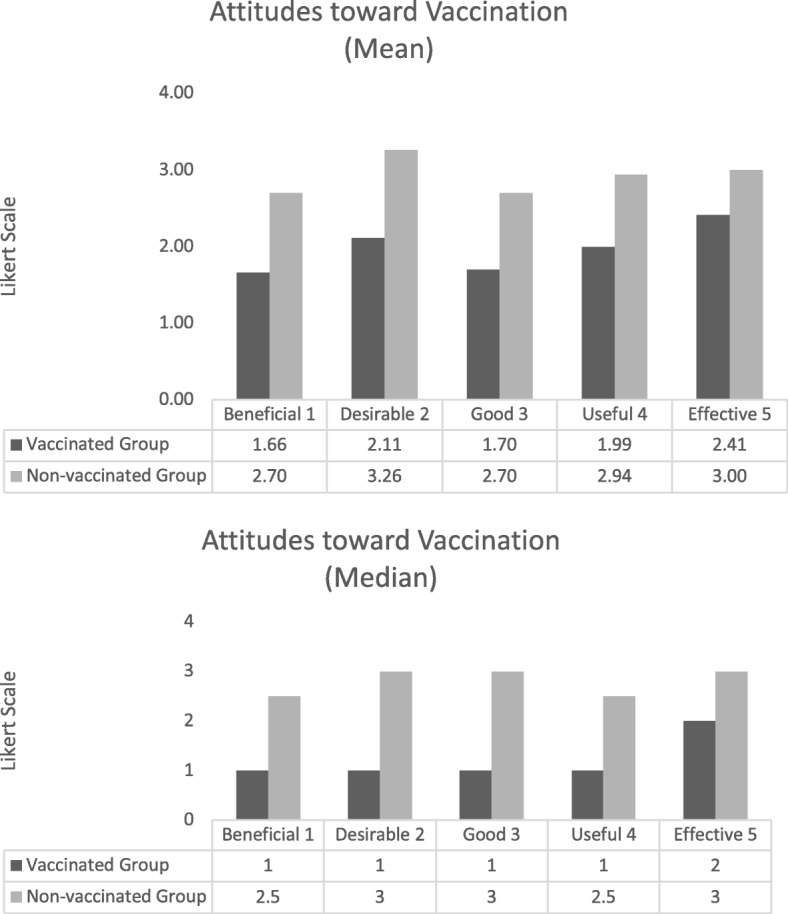
Attitudes toward HPV vaccination. Legend: Responses are based on a 1 to 7 Likert scale, where a 1 indicates a positive attitude. The scales were rated as ^1^ Beneficial to Harmful; ^2^ Desirable to Undesirable; ^3^ Good to Bad; ^4^ Useful to Worthless; ^5^ Effective to Ineffective. *P*-value based on the differences in means

Overall, the participants in this study had positive perceptions about HPV vaccination, though vaccinated women had more favourable views toward the vaccine. Figure 2 illustrates the differences based on five Likert scales, which combined, provided a cumulative value to describe an overall attitude towards receiving the vaccine.

Sub-analyses of overall attitudes towards the vaccine series between the vaccinated and unvaccinated groups indicated that these results did not significantly differ among ethnicities, with the exception of vaccinated white women, who had significantly more positive views towards accepting the vaccine than white non-vaccinated women (*p* < 0.0001). Both Canadian-born and immigrant vaccinated women also had significantly more favourable views than their non-vaccinated counterparts (*p* < 0.0001 and *p* = 0.04, respectively).

### Barriers to HPV vaccination

The cohort of unvaccinated women was asked a series of questions regarding the barriers they may have faced regarding HPV vaccination. Of the 12 possible barriers presented, the most influential obstacles were a lack of knowledge about the vaccine itself, its potential side effects and cost (Table 2). Only a fraction of the group (3.9%) was willing to pay the actual cost of the vaccine series, which can vary from $100 per dose at its cheapest to $175 per dose. Slightly more than half the women in the non-vaccinated group (55.1%) were willing to pay up to $100 for the entire vaccine series, while 10.7% of this group was unwilling to pay regardless of cost.

**Table 2:** Reported barriers to HPV Vaccination

Barriers	N non-vaccinated group (*n* = 205)	%
The vaccine costs too much		
Influential	90	45.69
Neutral	24	12.18
Not Influential	60	30.46
N/A^a^	23	11.68
The vaccine is not covered by my health insurance		
Influential	78	39.59
Neutral	25	12.69
Not Influential	60	30.46
N/A	34	17.26
I don’t know enough about the vaccine		
Influential	107	54.31
Neutral	20	10.15
Not Influential	49	24.87
N/A	21	10.66
I don’t know enough about the vaccine’s potential side effects		
Influential	126	66.64
Neutral	18	9.09
Not Influential	37	18.69
N/A	17	8.59
I don’t know if the vaccine works		
Influential	95	47.98
Neutral	35	17.68
Not Influential	53	26.77
N/A	15	7.58

^a^N/A: the participant does not think the barrier is applicable to her situation

Although not influential for the majority of the unvaccinated cohort, the ideas of not requiring the vaccine because the respondent was either in a monogamous relationship or was not currently sexually active were reported as influential for 24 and 29% of unvaccinated participants, respectively.

### Multivariable regression

The multivariable model compared two subpopulations in the unvaccinated cohort: those who intended to be vaccinated at some point in the future and those who did not.

The univariable analyses indicated that women who intended to be vaccinated had a more positive attitude towards HPV vaccination (lower overall mean score) and were influenced by the vaccine cost and vaccine potential to protect against cervical cancer. Participants who intended to be vaccinated were less likely to perceive cost as a barrier to vaccination. Social norms were particularly influential for the women who intended to be vaccinated, including a belief that if they received a recommendation from a doctor or a parent, or had friends who were getting or who had received the vaccine, they would be more likely to seek out the vaccine. In the final multivariate logistic regression model, only a more positive overall attitude to HPV vaccination and doctor recommendation were significant (Table 3).

**Table 3:** Comparison of respondents who intended and did not intend to be vaccinated

Independent Variables:	Odds ratio for intention to be vaccinated (*n* = 99), versus no intention to be vaccinated (*n* = 105)			
	Univariate Analysis		Multivariate Analysis^£^	
	OR	95% CI	OR	95% CI
More negative attitude to HPV vaccination	0.49***	[0.39, 0.63]	0.50**	[0.35, 0.71]
Less influenced by vaccine cost	1.86*	[1.01, 3.40]	1.10	[0.47, 2.58]
Do not know enough about the vaccine	0.75	[0.41, 1.37]	1.20	[0.49, 2.92]
Doctor recommendation	5.21***	[2.45, 11.09]	4.48**	[1.42, 14.13]
Friends got it/were going to get the vaccine	6.05***	[2.78, 13.13]	2.41	[0.94, 6.19]
Susceptibility to HPV	1.50	[0.81, 2.76]	0.73	[0.28, 1.91]
Parent recommendation	2.31*	[1.23. 4.35]	0.70	[0.26, 1.86]
Protect me from developing cervical cancer	17.35***	[2.23, 135.09]	1.08	[0.09, 12.45]
Increased age (per year)	0.94	[0.81, 1.09]	0.81	[0.65, 1.00]
Ethnicity (White vs. not white)	0.51*	[0.29, 0.90]	0.50	[0.22, 1.15]

Note: Those who did not intend to be vaccinated were used as the reference category (*N* = 105)

* *p* < 0.05 ** *p* < 0.01 ***p < 0.0001

^£^Model fit: Hosmer and Lemeshow Goodness-of-fit Test, *p* = 0.75

## Discussion

The prevalence of HPV vaccination in this study of undergraduate university women was 49%. Both vaccinated and non-vaccinated groups had positive overall attitudes toward HPV vaccination. The biggest barriers to HPV vaccination found in this study revolved around lack of knowledge about the vaccine and its potential side effects. Cost was an additional important barrier raised by the survey.

The vaccination prevalence in this study is comparable to the few other studies done in similar populations, though these studies were mostly conducted between 2008 and 2010, while our study was conducted in 2013. A study conducted in New York had a prevalence of vaccine series initiation of 56% [20], while students at a Midwestern university in the United States reported a vaccine series initiation prevalence of 49% [24]. In Marseilles, secondary and university students reported a prevalence of 35.4% [25]. These studies found that 70 to 80% of vaccine initiators completed the entire vaccine series [20, 24, 25]. However, a study conducted at McGill University in Montreal, Quebec, Canada indicated a HPV vaccination prevalence of only 27.3% [23], which contrasts starkly with our study, while Quebec data from 2010 to 2011 indicated vaccination coverage rates of grade four and nine girls surpassed 75% [26]. This contrast is likely due to data collection timing, as women at McGill University were not eligible for the free vaccine in elementary or secondary school prior to attending university, indicating the potential need for catch-up vaccination. The present study revealed that a sizeable proportion of unvaccinated women would be interested in receiving the vaccine at some point, which is comparable to the study in Montreal [23]. In the Canadian National Immunization Coverage survey in adults in 2012, 12.2% of sampled adults younger than 30 reported that they had received an HPV vaccination [27]. In 2014, 45% of females aged 18–26 and 85 aged 27–45 reported having been vaccinated with at least one dose of the HPV vaccine [28]. These findings indicate the importance of studies for young Canadian women, for whom a gap in vaccination coverage is evident.

A report from Public Health Ontario on immunization coverage in students found during the 2013–2014 school year, the proportion of vaccination coverage for meningococcus and hepatitis B was 77.5 and 71.5% respectively in 12-year-olds (age of vaccination), while HPV vaccine uptake was only 61.5% in 13-year-old girls (age of vaccination) [29]. This indicates a discrepancy in vaccine uptake in school-based vaccination programs, particularly for HPV and the ongoing relevance of catch-up programs, to cover nearly 40% of girls who are not vaccinated in school.

The Montreal study [23] also explored the relationship of a range of predictors to HPV vaccination intention, finding that negative perceived health consequences, doctor recommendation, attitudes and subjective norms were significant in multivariate analyses comparing unvaccinated women who did not intend to be vaccinated with those who did. Our study found similar results, with overall attitude towards the vaccine and doctor recommendation being significant in the final model. A systematic review assessing the factors associated with HPV vaccination in young women also found that positive vaccine attitudes were related to vaccine uptake [30]. This more favourable view among vaccinated versus non-vaccinated women could be due to increased knowledge about HPV infection, vaccination and vaccine safety, as those with more knowledge were more likely to be vaccinated [30–32].

The main barriers identified by our survey surrounded the topic of lack of vaccine knowledge. Unlike more established vaccines, the long-term side effects of the HPV vaccines are not widely known. These results are not unique to this study, as others also indicated that vaccine novelty, not knowing enough about it or its side effects, are among the chief reasons for university-aged women to not receive the vaccine [33, 34]. There are ongoing studies assessing the safety of the HPV vaccines following their implementation in diverse jurisdictions [35, 36]. Whilst mild and transient local reactions and systemic effects are commonly reported after vaccination, there is no evidence of a significant association with serious adverse effects, more than a decade after being approved for use in many jurisdictions. This knowledge, however, needs to be clearly communicated to potential vaccine recipients so that they have relevant information available to them on the balance of benefits and potential harms. This emphasizes the importance of knowledge translation strategies, including education by healthcare practitioners and shared decision making [35, 37, 38]. Other factors observed by these studies include being in a monogamous relationship, uncertainty about health insurance coverage and cost [33, 34], which were also all noted in our study. In our study, vaccine cost was reported to be an influential barrier in nearly half of the non-vaccinated group. Although many of these women stated that they wished to be vaccinated in the future, the cost of this vaccine series may be part of the reason why they want to defer vaccination to the future, instead of having it immediately. Most women in the unvaccinated group had a favourable opinion of the vaccine, so cost may not have had a large influence on their perception of the vaccine itself, but instead it may play a role in the complexity of prompting attitude and intention into receiving the vaccine.

One way to address barriers related to HPV vaccine knowledge is through a discussion with a family physician or gynaecologist; these providers are prepared to address most questions or concerns about HPV vaccination. The importance of education and health literacy has been highlighted in the literature for various health concerns. Without sufficient knowledge about cervical cancer and the role HPV plays, high vaccination rates may not be achieved. A study in the southern United States noted low vaccination rates among Hispanic women compared to the national rate. Institution of an education program about HPV vaccination and cervical cancer prevention found that knowledge increased after the program and the clinic’s HPV vaccination rates tripled [39]. Further, provincial and federal health agencies should consider allowing nurse practitioners and pharmacists to administer the vaccine once someone has the prescription, thereby making receiving it more accessible.

Although many of the unvaccinated women in the study intended to get vaccinated in the future, only a small subset was willing to pay the cost of the vaccine series. This could be enough to prevent intention from becoming behaviour. Clearly cost is not a barrier that can be resolved through education and has been a concern for women in many other studies [33, 34, 40].

In Ontario, a HPV vaccination program was implemented in 2007 specifically for eighth grade girls (ninth grade girls were eligible for the vaccine in the first year of the program only) [41]. This restriction continued until 2012, when the program was expanded to include coverage for all girls up to grade 12, as well as girls from the 2007/2008 cohort, which could be received at a local public health unit [41]. Implications of this restriction are that now, women who were in higher grades at program initiation were not vaccinated and therefore not protected against these HPV strains. These women continue to meet Gardasil’s eligibility criteria, but the results of the present study suggest that they are impeded from seeking to be vaccinated by a variety of barriers.

Assessing attitudes about HPV infection, vaccination and cervical cancer prevention has a significant role in health promotion and highlights the need for ongoing education about these important health concerns. As seen in many studies, attitudes affect intention to be vaccinated against HPV, while barriers to vaccination include lack of knowledge. It has been reported that increased knowledge can result in people become more receptive to prevention and screening programs or interventions [42]. Our study indicates the ongoing potential role of education in this area and the desire women have for more knowledge regarding their own health care decisions. Our findings indicate that women consider health and the role that healthcare providers play in education and informed healthcare decisions to be important, while elucidating barriers that need to be addressed, not only to improve vaccine uptake but also to affect general health practices. This study highlights the ongoing need for education around HPV, vaccination and cervical cancer, as well as the significant role a catch-up vaccination program can play specifically in this population.

Study strengths include its large and diverse sample. As the questionnaire was created anew, it was appropriate for the population studied. Additionally, the questionnaire was bilingual English/French, eliminating bias that could be introduced from neglecting a sizeable portion of the student population. Finally, this survey included an interesting sample of women: those who were offered the vaccine in schools after it had been introduced in 2007 and those who, due to age requirements, would not have been offered the vaccine.

The main limitation of this study was its response rate of 17%. Nonetheless, this study demonstrated the same proportion of vaccinated women as provincial statistics. Another limitation was the use of self-reported information, which can lead to recall and reporting errors. However, since this was a study of young educated women, this bias is unlikely to strongly influence the results of the study. Another consideration is that while intention to be vaccinated is a predictor of vaccine uptake, it is not the only predictor. Because of the complexity surrounding HPV vaccination uptake, including the perceptions of stigma related to the nature of HPV transmission, the requirement of multiple doses and vaccine cost, the constructs included in the TPB may be limited in fully explaining vaccination uptake.

## Conclusions

This study suggests that nearly half of the unvaccinated women surveyed would be interested in receiving the vaccine in the future, supporting the feasibility of a catch-up HPV vaccination program targeting a similar population. Although cost is not the main barrier preventing HPV vaccination in this population, it is amongst the foremost barriers. By increasing the financial accessibility of the vaccine, as well as focusing on further educating doctors, parents and women themselves on the purpose and benefits of the HPV vaccines, it would seem highly feasible to increase the prevalence of HPV vaccination in Canadian women substantially.

## Additional files

Additional file 1: This is a PDF of the English version of the questionnaire that was administered to the study participants. The data presented in the study was collected and analysed from these questionnaires. (PDF 195 kb)

Additional file 2: This is a PDF of the French version of the questionnaire that was administered to the study participants. The data presented in the study was collected and analysed from these questionnaires. (PDF 189 kb)

Additional file 3: The database for the information collected from the survey has also been included as an xls extension. This is the raw data collected from the study questionnaires that was used for analysis. (XLS 501 kb)

## Abbreviations

CI: Confidence interval
HPV: Human papillomavirus
OR: Odds Ratio
TPB: Theory of Planned Behaviour
