# Supplementary material for: Attitudes of undergraduate university women towards HPV vaccination: a cross-sectional study in Ottawa, Canada
Source: BMC Womens Health. 2018 Aug 2;18:134. doi: 10.1186/s12905-018-0622-0 (PMC6090914; doi:10.1186/s12905-018-0622-0)
Supplement: Supplementary file 1 — This is a PDF of the English version of the questionnaire that was administered to the study participants. The data presented in the study was collected and analysed from these questionnaires. (PDF 195 kb) [file 12905_2018_622_MOESM1_ESM.pdf]

English Questionnaire

The survey you are about to do is about your attitudes about the Human Papillomavirus (HPV) vaccine. This survey can be completed regardless of whether or not you have ever received the vaccine. Please respond as honestly as possible.

The Human Papillomavirus (HPV) is a virus that is responsible for a variety of genital infections. It is also the primary cause of cervical cancer, which is one of the most common types of cancer in women worldwide. The Papanicolaou test, more commonly known as a Pap test or a Pap smear, is used to detect cervical abnormalities and pre-cancerous or cancerous lesions.

1) Are you female or male?
- ☐ Female
- ☐ Male (Will be redirected to the thank you page of the survey)

2) Have you been vaccinated with at least 1 dose of an HPV vaccine?
- ☐ Yes (Will be directed to the next question)
- ☐ No (Will be directed to Question 16)

3) How many doses of the HPV vaccine have you had?
- ☐ 1
- ☐ 2
- ☐ 3
- ☐ 4 or more
- ☐ I'm not sure

Section 1a: Attitude about HPV vaccines and getting the HPV vaccine

In this section, you will be asked about your opinions of the HPV vaccine. Though some of the questions may seem similar, please answer each of them. The questions in this section are mostly based on a 1 to 7 rating scale. For each statement, indicate the number on the scale that you think best reflects your opinion or belief.

4) To me, getting vaccinated against HPV was…

Beneficial	1	2	3	4	5	6	7	Harmful
Undesirable	1	2	3	4	5	6	7	Desirable
Good	1	2	3	4	5	6	7	Bad
Worthless	1	2	3	4	5	6	7	Useful
Effective	1	2	3	4	5	6	7	Ineffective

5) Getting the HPV vaccine has decreased my risk of getting genital warts.

Extremely likely	1	2	3	4	5	6	7	Extremely unlikely

6) Decreasing my risk of getting genital warts is…

Beneficial	1	2	3	4	5	6	7	Harmful
Undesirable	1	2	3	4	5	6	7	Desirable
Good	1	2	3	4	5	6	7	Bad

7) Getting the HPV vaccine has decreased my risk of getting cervical cancer.

Extremely likely	1	2	3	4	5	6	7	Extremely unlikely

8) Decreasing my risk of getting cervical cancer is…

Beneficial	1	2	3	4	5	6	7	Harmful
Undesirable	1	2	3	4	5	6	7	Desirable
Good	1	2	3	4	5	6	7	Bad

9) Getting the HPV vaccine has decreased my need for cervical cancer screening in the future.

Extremely likely	1	2	3	4	5	6	7	Extremely unlikely

10) Cervical cancer screening is…

Beneficial	1	2	3	4	5	6	7	Harmful
Undesirable	1	2	3	4	5	6	7	Desirable
Good	1	2	3	4	5	6	7	Bad

11) Vaccinating all eligible girls/women against HPV, if they are not sexually active is…

Beneficial	1	2	3	4	5	6	7	Harmful
Undesirable	1	2	3	4	5	6	7	Desirable
Good	1	2	3	4	5	6	7	Bad
Important	1	2	3	4	5	6	7	Unimportant

12) Vaccinating all eligible girls/women against HPV, if they are sexually active is…

Beneficial	1	2	3	4	5	6	7	Harmful
Undesirable	1	2	3	4	5	6	7	Desirable
Good	1	2	3	4	5	6	7	Bad
Important	1	2	3	4	5	6	7	Unimportant

13) I intend to finish my HPV vaccine series

- ☐ Yes, as per the vaccine schedule
- ☐ Yes, but it will take longer than the scheduled time frame
- ☐ I have already gotten all my vaccine doses
- ☐ No (Reason:_______________)

14) Please indicate the extent to which you agree or disagree with the following statements.

a) Getting the HPV vaccine has changed my intentions to get cervical cancer screening

Strongly Agree	1	2	3	4	5	Strongly Disagree

b) I intend to get cervical cancer screening in the future

Strongly Agree	1	2	3	4	5	Strongly Disagree

c) HPV vaccination has not affected my decision about cervical cancer screening

Strongly Agree	1	2	3	4	5	Strongly Disagree

<u>Section 2a: Motivations and Barriers</u>

In this section we would like to ask you about what influenced your decision to get the HPV vaccine.

15) If you HAVE had at least one dose of the HPV vaccine, how influential each of the following factors was in making your decision:

My doctor recommended it	Very Influential	Somewhat Influential	Neutral	Not Very Influential	Not At All Influential	N/A
I saw advertisement(s) for it	Very Influential	Somewhat Influential	Neutral	Not Very Influential	Not At All Influential	N/A
My friend(s) got the vaccine or were going to get it	Very Influential	Somewhat Influential	Neutral	Not Very Influential	Not At All Influential	N/A
I think I am susceptible to HPV infection	Very Influential	Somewhat Influential	Neutral	Not Very Influential	Not At All Influential	N/A
There is a history of cervical abnormalities in my family	Very Influential	Somewhat Influential	Neutral	Not Very Influential	Not At All Influential	N/A
I know someone who has/had cervical cancer	Very Influential	Somewhat Influential	Neutral	Not Very Influential	Not At All Influential	N/A
My parents recommended it	Very Influential	Somewhat Influential	Neutral	Not Very Influential	Not At All Influential	N/A
My friend(s) recommended it	Very Influential	Somewhat Influential	Neutral	Not Very Influential	Not At All Influential	N/A
It was covered by my health insurance	Very Influential	Somewhat Influential	Neutral	Not Very Influential	Not At All Influential	N/A
It was offered to me through my school (elementary, middle or high school)	Very Influential	Somewhat Influential	Neutral	Not Very Influential	Not At All Influential	N/A
Most girls/women my age were getting the vaccine	Very Influential	Somewhat Influential	Neutral	Not Very Influential	Not At All Influential	N/A

16) If you HAVE had a least one dose of the HPV vaccine, indicate the extent to which the following potential benefits to HPV vaccination influenced your decision to get the vaccine.

It may protect me against HPV infection	Very Influential	Somewhat Influential	Neutral	Not Very Influential	Not At All Influential	N/A
It may protect me from getting genital warts	Very Influential	Somewhat Influential	Neutral	Not Very Influential	Not At All Influential	N/A
It may protect me from developing cervical cancer	Very Influential	Somewhat Influential	Neutral	Not Very Influential	Not At All Influential	N/A

[Will be directed to section 3]

<u>Section 1b: Attitude about HPV vaccines and getting the HPV vaccine</u>

In this section, you will be asked about your opinions of the HPV vaccine. Though some of the questions may seem similar, please answer each of them. The questions in this section are mostly based on a 1 to 7 rating scale. For each statement, indicate the number on the scale that you think best reflects your opinion or belief.

17) To me, getting vaccinated against HPV would be…

Beneficial	1	2	3	4	5	6	7	Harmful
Undesirable	1	2	3	4	5	6	7	Desirable
Good	1	2	3	4	5	6	7	Bad
Worthless	1	2	3	4	5	6	7	Useful
Effective	1	2	3	4	5	6	7	Ineffective

18) Getting the HPV vaccine will decrease my risk of getting genital warts.

Extremely likely	1	2	3	4	5	6	7	Extremely unlikely

19) Decreasing my risk of getting genital warts is…

Beneficial	1	2	3	4	5	6	7	Harmful
Undesirable	1	2	3	4	5	6	7	Desirable
Good	1	2	3	4	5	6	7	Bad

20) Getting the HPV vaccine will decrease my risk of getting cervical cancer.

Extremely likely	1	2	3	4	5	6	7	Extremely unlikely

21) Decreasing my risk of getting cervical cancer is…

Beneficial	1	2	3	4	5	6	7	Harmful
Undesirable	1	2	3	4	5	6	7	Desirable
Good	1	2	3	4	5	6	7	Bad

22) Getting the HPV vaccine will decrease my need for cervical cancer screening in the future.

Extremely likely	1	2	3	4	5	6	7	Extremely unlikely

23) Cervical cancer screening is…

Beneficial	1	2	3	4	5	6	7	Harmful
Undesirable	1	2	3	4	5	6	7	Desirable
Good	1	2	3	4	5	6	7	Bad

24) Vaccinating all eligible girls/women against HPV, if they are not sexually active is…

Beneficial	1	2	3	4	5	6	7	Harmful
Undesirable	1	2	3	4	5	6	7	Desirable
Good	1	2	3	4	5	6	7	Bad
Important	1	2	3	4	5	6	7	Unimportant

25) Vaccinating all eligible girls/women against HPV, if they are sexually active is…

Beneficial	1	2	3	4	5	6	7	Harmful
Undesirable	1	2	3	4	5	6	7	Desirable
Good	1	2	3	4	5	6	7	Bad
Important	1	2	3	4	5	6	7	Unimportant

26) Please indicate the extent to which you agree or disagree with the following statements.

a) I intend to get the HPV vaccine in the next 12 months

Strongly Agree 1 2 3 4 5 Strongly Disagree

b) I do not intend to get the HPV vaccine

Strongly Agree 1 2 3 4 5 Strongly Disagree

c) I plan on getting the HPV vaccine at some point

Strongly Agree 1 2 3 4 5 Strongly Disagree

27) Please indicate the extent to which you agree or disagree with the following statements.

a) Getting the HPV vaccine would change my intentions to get cervical cancer screening

Strongly Agree 1 2 3 4 5 Strongly Disagree

b) I intend to get cervical cancer screening in the future

Strongly Agree 1 2 3 4 5 Strongly Disagree

c) HPV vaccination would not affect my decision about cervical cancer screening

Strongly Agree 1 2 3 4 5 Strongly Disagree

Section 2b: Motivations and Barriers

In this section we would like to ask you about what has influenced your decision to not get the HPV vaccine.

28) If you have NOT had at least one dose of the HPV vaccine, indicate how influential each of the following factors was in making your decision:

I have not heard of the vaccine	Very Influential	Somewhat Influential	Neutral	Not Very Influential	Not At All Influential	N/A
I don't know where the vaccine is offered	Very Influential	Somewhat Influential	Neutral	Not Very Influential	Not At All Influential	N/A
The vaccine costs too much	Very Influential	Somewhat Influential	Neutral	Not Very Influential	Not At All Influential	N/A
The vaccine is not covered by my health insurance	Very Influential	Somewhat Influential	Neutral	Not Very Influential	Not At All Influential	N/A
I'm in a monogamous relationship and therefore don't need the vaccine	Very Influential	Somewhat Influential	Neutral	Not Very Influential	Not At All Influential	N/A
I'm not currently having sex and therefore don't need the vaccine	Very Influential	Somewhat Influential	Neutral	Not Very Influential	Not At All Influential	N/A
My parents don't want me to get the vaccine	Very Influential	Somewhat Influential	Neutral	Not Very Influential	Not At All Influential	N/A
I don't know enough about the vaccine	Very Influential	Somewhat Influential	Neutral	Not Very Influential	Not At All Influential	N/A
I don't know enough about the vaccine's potential side effects	Very Influential	Somewhat Influential	Neutral	Not Very Influential	Not At All Influential	N/A
I don't know if the vaccine works	Very Influential	Somewhat Influential	Neutral	Not Very Influential	Not At All Influential	N/A
I am concerned that the vaccine will hurt	Very Influential	Somewhat Influential	Neutral	Not Very Influential	Not At All Influential	N/A
I already have HPV	Very Influential	Somewhat Influential	Neutral	Not Very Influential	Not At All Influential	N/A

29) If you have NOT yet had at least one dose of the HPV vaccine, indicate how influential each of the following factors would be in making your decision to receive the HPV vaccine.

My doctor recommended it	Very Influential	Somewhat Influential	Neutral	Not Very Influential	Not At All Influential	N/A
I saw advertisement(s) for it	Very Influential	Somewhat Influential	Neutral	Not Very Influential	Not At All Influential	N/A
My friend(s) got the vaccine or were going to get it	Very Influential	Somewhat Influential	Neutral	Not Very Influential	Not At All Influential	N/A
I think I am susceptible to HPV infection	Very Influential	Somewhat Influential	Neutral	Not Very Influential	Not At All Influential	N/A
There is a history of cervical abnormalities in my family	Very Influential	Somewhat Influential	Neutral	Not Very Influential	Not At All Influential	N/A
I know someone who has/had cervical cancer	Very Influential	Somewhat Influential	Neutral	Not Very Influential	Not At All Influential	N/A
My parents recommended it	Very Influential	Somewhat Influential	Neutral	Not Very Influential	Not At All Influential	N/A
My friend(s) recommended it	Very Influential	Somewhat Influential	Neutral	Not Very Influential	Not At All Influential	N/A
It was free or covered by my health insurance	Very Influential	Somewhat Influential	Neutral	Not Very Influential	Not At All Influential	N/A
It was offered to me through my school (elementary, middle or high school)	Very Influential	Somewhat Influential	Neutral	Not Very Influential	Not At All Influential	N/A
Vaccine cost	Very Influential	Somewhat Influential	Neutral	Not Very Influential	Not At All Influential	N/A
Most girls/women my age were getting the vaccine	Very Influential	Somewhat Influential	Neutral	Not Very Influential	Not At All Influential	N/A
It may protect me against HPV infection	Very Influential	Somewhat Influential	Neutral	Not Very Influential	Not At All Influential	N/A
It may protect me from getting genital warts	Very Influential	Somewhat Influential	Neutral	Not Very Influential	Not At All Influential	N/A
It may protect me from developing cervical cancer	Very Influential	Somewhat Influential	Neutral	Not Very Influential	Not At All Influential	N/A

30) If your health insurance did not cover it, what is the most you would be willing to pay for the HPV vaccine series?

- ☐ $0
- ☐ More than $0, but less than or equal to $100
- ☐ $200
- ☐ $300
- ☐ $400
- ☐ Greater than or equal to $500
- ☐ I would not get the vaccine

Section 3: Vaccine Acceptability and Safe Sex practices
In this section we would like to know how this vaccine could alter your future health and safe sex practices.

31) Getting the HPV vaccine has/would make me less likely to get regular Pap smears.
- ☐ Strongly Agree
- ☐ Agree
- ☐ Neutral
- ☐ Disagree
- ☐ Strongly Disagree

32) Assuming that you have had or were going to get the HPV vaccine, please indicate the extent to which you agree or disagree with the following statements about safe sex behaviours.
Complete the statement:
"After getting vaccinated against HPV ______________________*"*

I think that condom use during sex is less necessary	Strongly Agree	Agree	Neutral	Disagree	Strongly Disagree
I feel it is still just as important to have as few sexual partners as possible	Strongly Agree	Agree	Neutral	Disagree	Strongly Disagree
I feel that it is not as important to talk to my sex partners about safe sex	Strongly Agree	Agree	Neutral	Disagree	Strongly Disagree
I think it is still as important to use a condom everytime I have sex	Strongly Agree	Agree	Neutral	Disagree	Strongly Disagree
I will be less worried about having unprotected sex	Strongly Agree	Agree	Neutral	Disagree	Strongly Disagree

33) Has/would receiving the HPV vaccine affect how often you would use a form of birth control or contraception during sex, compared to before you received the vaccine?
- ☐ Yes, I would use it more frequently
- ☐ Yes, I would use it less frequently
- ☐ No, I would not change the frequency with which I used birth control/contraception methods
- ☐ Don't know
- ☐ N/A

Section 4: Knowledge of HPV and Cervical Cancer

There are 2 different HPV vaccines available to women. These vaccines, Gardasil® and Cervarix® both provide protection against the most common strains of HPV that cause cervical cancer. Gardasil® also provides protection against some additional types of HPV that cause genital warts but not cancer.

In this section, we would like to know how familiar you are with HPV and cervical cancer facts.

34) Indicate whether you think each of the following statements about HPV is true or false.

**HPV is primarily sexually transmitted**	True	False
**HPV is the most common STI (sexually transmitted infection)**	True	False
**HPV only affects women**	True	False
**Most people infected with HPV do not show any signs or symptoms of infections**	True	False
**HPV vaccination is only effective if you are not sexually active**	True	False
**The highest rate of HPV infection is in adults <25 years of age**	True	False
**HPV infection can lead to cervical cancer**	True	False
**3 HPV vaccine doses are required to have the fullest protection**	True	False
**At least 1 in 2 women is diagnosed with HPV in her lifetime**	True	False
**HPV vaccination protects you from all HPV-related infections**	True	False
**The most common sign of HPV infection is genital warts**	True	False
**HPV vaccination is only effective for women**	True	False
**HPV infection has been associated with several types of cancer**	True	False
**There are 2 HPV strains that account for 70% of cervical cancers**	True	False
**HPV is the only proven cause of cervical cancer**	True	False
**Pap tests are unnecessary if women are vaccinated against HPV**	True	False

35) Before today, were you aware that there are vaccines available that can help prevent cervical cancer and other HPV-related infections?

- ☐ Yes
- ☐ No

36) Before today, were you aware that there were 2 different vaccines available against HPV?

- ☐ Yes
- ☐ No

Section 5: Demographics

In this final section, we would like to know some background information.

37) Which Faculty are you in?

- ☐ Arts
- ☐ Science
- ☐ Engineering
- ☐ Health Sciences
- ☐ Other (please specify)___________________

38) How old are you?

- ☐ Less than 18 years
- ☐ 18 years
- ☐ 19 years
- ☐ 20 years
- ☐ 21 years
- ☐ 22 years
- ☐ 23 years
- ☐ 24 years
- ☐ 25 years
- ☐ More than 25 years

39) You may belong to one or more racial or cultural groups on the following list. What are your ethnic or cultural origins? *(Check all that apply)*:

- ☐ White
- ☐ South Asian (e.g. East Indian, Pakistani, Sri Lankan etc)
- ☐ Chinese
- ☐ Black
- ☐ Filipino
- ☐ Latin American
- ☐ Arab
- ☐ South-east Asian (e.g. Vietnamese, Cambodian, Malaysian, Laotian, etc)
- ☐ West Asian (e.g. Iranian, Afghani, etc)
- ☐ Korean
- ☐ Japanese
- ☐ Aboriginal (includes First Nation/Inuit/Métis)
- ☐ Other (please specify)___________________

40) Prior to starting at the University of Ottawa, where did you live?

- ☐ Alberta
- ☐ British Columbia
- ☐ Manitoba
- ☐ New Brunswick
- ☐ Newfoundland and Labrador
- ☐ Nova Scotia

- ☐ Ontario
- ☐ Prince Edward Island
- ☐ Quebec
- ☐ Saskatchewan
- ☐ Northwest Territory
- ☐ Nunavut
- ☐ Yukon
- ☐ Other country (please specify)____________________

41) Please select the answer that best corresponds to your current health insurance plan, in addition to provincial healthcare (OHIP, RAMQ).

- ☐ I am covered by my parent(s) or guardian's health insurance plan
- ☐ I am covered by the university's health insurance plan
- ☐ I have my own private health insurance plan
- ☐ I am covered by my spouse's health insurance plan
- ☐ I do not have a health insurance plan

42) Have you ever had sex (oral, vaginal, or anal) with a male partner?

- ☐ Yes
- ☐ No

43) Have you ever had a Pap smear?

- ☐ Yes, more than once
- ☐ Yes, once
- ☐ No
- ☐ Don't know

44) Have you ever had an abnormal Pap smear?

- ☐ Yes
- ☐ No
- ☐ Don't know

45) Are you up to date on your regular vaccines (i.e. have you had your booster shots)?

- ☐ Yes
- ☐ No
- ☐ Don't know

46) Are you currently using some form of birth control/contraception?

- ☐ Yes
- ☐ No

47) Have you ever been told by a healthcare professional that you had an HPV infection?

- Yes
- No
- Don't know

48) Have you ever been told by a healthcare professional that you had an STI (sexually transmitted infection) other than HPV?

- Yes
- No
- Don't know

49) Were you born in Canada?

- Yes
- No
