# Supplementary material for: Attitudes of undergraduate university women towards HPV vaccination: a cross-sectional study in Ottawa, Canada
Source: BMC Womens Health. 2018 Aug 2;18:134. doi: 10.1186/s12905-018-0622-0 (PMC6090914; doi:10.1186/s12905-018-0622-0)
Supplement: Supplementary file 2 — This is a PDF of the French version of the questionnaire that was administered to the study participants. The data presented in the study was collected and analysed from these questionnaires. (PDF 189 kb) [file 12905_2018_622_MOESM2_ESM.pdf]

French Questionnaire

Ce sondage vise à récolter vos opinions et vos perceptions sur la vaccination contre le virus du papillome humain (VPH). Vous pouvez remplir ce sondage que vous ayez reçu ou non le vaccin contre le VPH. S'il vous, veuillez répondre le plus honnêtement possible.

Le VPH est un virus qui cause une variété de maladies génitales. De plus, ce virus est la cause principale du cancer du col de l'utérus, l'un des cancers les plus répandus chez les femmes dans le monde entier. Le test de Papanicolaou, généralement connu comme un test de Pap, est utilisé pour détecter les anomalies du col de l'utérus et des lésions associées avec le cancer.

1) Êtes-vous une femme ou un homme?
- ☐ Femme
- ☐ Homme

2) Êtes-vous vaccinée avec au moins une dose du vaccin contre le VPH?
- ☐ Oui
- ☐ Non

3) Combien doses du vaccin contre le VPH avez-vous reçu?
- ☐ 1
- ☐ 2
- ☐ 3
- ☐ 4 ou plus
- ☐ Je ne suis pas certaine

Section 1a : Attitudes au sujet des vaccins contre le VPH et recevoir ce vaccin

Cette section porte sur vos opinions par rapport à la vaccination contre le VPH. Même si les questions ont l'air d'être similaires, veuillez répondre à chacune d'entre elles. La plupart des questions ci-dessous ont été développées sur une échelle de 7 points. Pour chaque question, indiquer le numéro dont qui correspond le plus à vos opinions ou vos croyances.

4) D'après moi, recevoir la vaccination contre le VPH était…

Bénéfique	1	2	3	4	5	6	7	Nuisible
Indésirable	1	2	3	4	5	6	7	Désirable
Bonne	1	2	3	4	5	6	7	Mauvaise
Inutile	1	2	3	4	5	6	7	Utile
Efficace	1	2	3	4	5	6	7	Inefficace

5) Recevoir le vaccin contre le VPH a réduit mon risque de développer des verrues génitales….

Très probable	1	2	3	4	5	6	7	Peu probable

6) Réduire mon risque de développer les verrues génitales est….

Bénéfique	1	2	3	4	5	6	7	Nuisible
Indésirable	1	2	3	4	5	6	7	Désirable
Bon	1	2	3	4	5	6	7	Mauvais

7) Recevoir le vaccin contre le VPH a réduit mon risque de développer le cancer du col de l'utérus…

Très probable	1	2	3	4	5	6	7	Peu probable

8) Réduire mon risque de développer le cancer du col de l'utérus est…

Bénéfique	1	2	3	4	5	6	7	Nuisible
Indésirable	1	2	3	4	5	6	7	Désirable
Bon	1	2	3	4	5	6	7	Mauvais

9) Recevoir le vaccin contre le VPH a réduit la nécessité d'avoir un test de dépistage pour le cancer du col de l'utérus…

Très probable	1	2	3	4	5	6	7	Peu probable

10) Le dépistage du cancer du col de l'utérus est…

Bénéfique	1	2	3	4	5	6	7	Nuisible
Indésirable	1	2	3	4	5	6	7	Désirable
Bon	1	2	3	4	5	6	7	Mauvais

11) La vaccination de toutes les filles/femmes contre le VPH, si elles ne sont pas sexuellement actives, est…

Bénéfique	1	2	3	4	5	6	7	Nuisible
Indésirable	1	2	3	4	5	6	7	Désirable
Bonne	1	2	3	4	5	6	7	Mauvaise
Importante	1	2	3	4	5	6	7	Non importante

12) La vaccination de toutes les filles/femmes contre le VPH, si elles sont sexuellement actives, est…

Bénéfique	1	2	3	4	5	6	7	Nuisible
Indésirable	1	2	3	4	5	6	7	Désirable
Bonne	1	2	3	4	5	6	7	Mauvaise
Importante	1	2	3	4	5	6	7	Non importante

13) J'ai l'intention de finir la série des vaccins contre le VPH

- ☐ Oui, conformément à l'horaire indiqué pour la série.
- ☐ Oui, mais je le ferai en plus de temps qu'indiqué pour la série de vaccins.
- ☐ J'ai déjà reçu toutes les doses de vaccin.
- ☐ Non (Raison(s): ________________________)

14) SVP indiquez à quel point vous êtes d'accord avec les déclarations suivantes.

a) Recevoir le vaccin contre le VPH a changé mes intentions de recevoir ou non un test de dépistage du cancer du col de l'utérus.

Fermement en accord	1	2	3	4	5	Fermement en désaccord

b) J'ai l'intention de recevoir un test de dépistage du cancer du col de l'utérus.

Fermement en accord	1	2	3	4	5	Fermement en désaccord

c) Le vaccin contre le VPH n’a pas affecté ma décision concernant le dépistage du cancer du col de l’utérus.

Fermement en accord 1 2 3 4 5 Fermement en désaccord

Section 2a : Motivations et obstacles
Dans cette section, nous vous demandons ce qui a influencé votre décision de recevoir le vaccin contre le VPH.

15) Si vous AVEZ reçu au moins une dose du vaccin contre le VPH, indiquez à quel point chacun de ces facteurs suivants a influencé votre décision.

Mon médecin me l'a recommandé.	Très influent	Un peu influent	Neutre	Pas très influent	Pas du tout influent	N/A
J'ai vu une (plusieurs) publicité(s) pour le vaccin.	Très influent	Un peu influent	Neutre	Pas très influent	Pas du tout influent	N/A
Une (des) amie(s) a (ont) reçu le vaccin ou avait(ent) l'intention de le recevoir.	Très influent	Un peu influent	Neutre	Pas très influent	Pas du tout influent	N/A
Je pense que je suis sujette à une infection de VPH	Très influent	Un peu influent	Neutre	Pas très influent	Pas du tout influent	N/A
Il y a des antécédents d'anomalies du col de l'utérus dans ma famille.	Très influent	Un peu influent	Neutre	Pas très influent	Pas du tout influent	N/A
Je connais quelqu'un qui a/avait le cancer du col de l'utérus.	Très influent	Un peu influent	Neutre	Pas très influent	Pas du tout influent	N/A
Mes parents me l'ont recommandé.	Très influent	Un peu influent	Neutre	Pas très influent	Pas du tout influent	N/A
Mon (mes) amie(s) me l'a (ont) recommandé.	Très influent	Un peu influent	Neutre	Pas très influent	Pas du tout influent	N/A
Le vaccin était couvert par mon assurance maladie.	Très influent	Un peu influent	Neutre	Pas très influent	Pas du tout influent	N/A
Le vaccin m'était offert à l'école (primaire, secondaire).	Très influent	Un peu influent	Neutre	Pas très influent	Pas du tout influent	N/A
La plupart des filles/femmes mon âge recevait le vaccin.	Très influent	Un peu influent	Neutre	Pas très influent	Pas du tout influent	N/A

16) Si vous AVEZ reçu au moins une dose du vaccin contre le VPH, veuillez indiquez à quel point les avantages potentiels suivants à la vaccination contre le VPH ont influencé votre décision de recevoir ce vaccin.

Le vaccin pourrait me protéger contre une infection de VPH.	Très influent	Un peu influent	Neutre	Pas très influent	Pas du tout influent	N/A
Le vaccin pourrait me protéger contre le développement des verrues génitales.	Très influent	Un peu influent	Neutre	Pas très influent	Pas du tout influent	N/A
Le vaccin pourrait me protéger contre le développement du cancer du col de l'utérus.	Très influent	Un peu influent	Neutre	Pas très influent	Pas du tout influent	N/A

Section 1b : Attitudes au sujet des vaccins contre le VPH et l'administration du vaccin
Cette section vous demande vos opinions par rapport à la vaccination contre le VPH. Même si les questions ont l'air d'être similaire, veuillez répondre à chacune d'entre elles. La plupart des questions ci-dessous ont été développées sur une échelle de 7 points. Pour chaque question, indiquer le numéro dont qui correspond le plus à vos opinions ou vos croyances.

17) D'après moi, recevoir la vaccination contre le VPH serait…

Bénéfique	1	2	3	4	5	6	7	Nuisible
Indésirable	1	2	3	4	5	6	7	Désirable
Bonne	1	2	3	4	5	6	7	Mauvaise
Inutile	1	2	3	4	5	6	7	Utile
Efficace	1	2	3	4	5	6	7	Inefficace

18) Recevoir le vaccin contre le VPH réduira mon risque de développer des verrues génitales…

Très probable	1	2	3	4	5	6	7	Peu probable

19) Réduire mon risque de développer les verrues génitales est….

Bénéfique	1	2	3	4	5	6	7	Nuisible
Indésirable	1	2	3	4	5	6	7	Désirable
Bon	1	2	3	4	5	6	7	Mauvais

20) Recevoir le vaccin contre le VPH réduira mon risque de développer le cancer du col de l'utérus…

Très probable	1	2	3	4	5	6	7	Peu probable

21) Réduire mon risque de développer le cancer du col de l'utérus est…

Bénéfique	1	2	3	4	5	6	7	Nuisible
Indésirable	1	2	3	4	5	6	7	Désirable
Bon	1	2	3	4	5	6	7	Mauvais

22) Recevoir le vaccin contre le VPH réduira la nécessité d'avoir un test de dépistage pour le cancer du col de l'utérus…

Très probable	1	2	3	4	5	6	7	Peu probable

23) Le dépistage du cancer du col de l'utérus est…

Bénéfique	1	2	3	4	5	6	7	Nuisible
Indésirable	1	2	3	4	5	6	7	Désirable
Bon	1	2	3	4	5	6	7	Mauvais

24) La vaccination de toutes les filles/femmes contre le VPH, si elles ne sont pas sexuellement actives, est…

Bénéfique	1	2	3	4	5	6	7	Nuisible
Indésirable	1	2	3	4	5	6	7	Désirable
Bonne	1	2	3	4	5	6	7	Mauvaise
Importante	1	2	3	4	5	6	7	Non importante

25) La vaccination de toutes les filles/femmes contre le VPH, si elles sont sexuellement actives, est…

Bénéfique	1	2	3	4	5	6	7	Nuisible
Indésirable	1	2	3	4	5	6	7	Désirable
Bonne	1	2	3	4	5	6	7	Mauvaise
Importante	1	2	3	4	5	6	7	Non importante

26) SVP indiquez à quel point vous êtes d'accord avec les déclarations suivantes.

a) J'ai l'intention de recevoir le vaccin contre le VPH durant les prochains 12 mois.
Fermement en accord 1 2 3 4 5 Fermement en désaccord

b) Je n'ai pas l'intention de recevoir le vaccin contre le VPH.
Fermement en accord 1 2 3 4 5 Fermement en désaccord

c) J'envisage de recevoir le vaccin contre le VPH à un moment.
Fermement en accord 1 2 3 4 5 Fermement en désaccord

27) SVP indiquez à quel point vous êtes d'accord avec les déclarations suivantes.

a) Recevoir le vaccin contre le VPH changera mes intentions envers le dépistage du cancer du col de l'utérus.
Fermement en accord 1 2 3 4 5 Fermement en désaccord

b) J'ai l'intention de recevoir un est de dépistage du cancer du col de l'utérus.
Fermement en accord 1 2 3 4 5 Fermement en désaccord

c) Le vaccin contre le VPH n'affectera pas ma décision envers le dépistage du cancer du col de l'utérus.
Fermement en accord 1 2 3 4 5 Fermement en désaccord

Section 2b : Motivations et obstacles
Dans cette section, nous vous demandons ce qui a influencé votre décision de recevoir le vaccin contre le VPH.

28) Si vous N'AVEZ PAS reçu au moins une dose du vaccin contre le VPH, indiquez à quel point chacun des facteurs suivants a influencé votre décision.

Je n'ai jamais entendu parler du vaccin.	Très influent	Un peu influent	Neutre	Pas très influent	Pas du tout influent	N/A
Je ne sais pas où ce vaccin est offert.	Très influent	Un peu influent	Neutre	Pas très influent	Pas du tout influent	N/A
Le vaccin est trop cher.	Très influent	Un peu influent	Neutre	Pas très influent	Pas du tout influent	N/A
Le vaccin n'est pas couvert par mon assurance de maladie.	Très influent	Un peu influent	Neutre	Pas très influent	Pas du tout influent	N/A
Je suis dans une relation monogame alors je n'ai pas besoin ce vaccin.	Très influent	Un peu influent	Neutre	Pas très influent	Pas du tout influent	N/A
En ce moment je ne suis pas sexuellement active alors je n'ai pas besoin ce vaccin.	Très influent	Un peu influent	Neutre	Pas très influent	Pas du tout influent	N/A
Mes parents ne veulent pas que je reçois ce vaccin.	Très influent	Un peu influent	Neutre	Pas très influent	Pas du tout influent	N/A
Je n'en connais pas assez sur ce vaccin.	Très influent	Un peu influent	Neutre	Pas très influent	Pas du tout influent	N/A
Je n'en connais pas assez sure les effets secondaires potentiels de ce vaccin.	Très influent	Un peu influent	Neutre	Pas très influent	Pas du tout influent	N/A
Je ne sais pas si ce vaccin fonctionne.	Très influent	Un peu influent	Neutre	Pas très influent	Pas du tout influent	N/A
J'ai peur que le vaccin me fasse mal.	Très influent	Un peu influent	Neutre	Pas très influent	Pas du tout influent	N/A
J'ai déjà le VPH.	Très influent	Un peu influent	Neutre	Pas très influent	Pas du tout influent	N/A

29) Si vous N'AVEZ PAS reçu au moins une dose du vaccin contre le VPH, indiquez à quel point chacun des facteurs suivants influencera votre décision.

Mon médecin me l'a recommandé.	Très influent	Un peu influent	Neutre	Pas très influent	Pas du tout influent	N/A
J'ai vu une (plusieurs) publicité(s) pour le vaccin.	Très influent	Un peu influent	Neutre	Pas très influent	Pas du tout influent	N/A
Une (des) amie(s) a (ont) reçu le vaccin ou avait(ent) l'intention de le recevoir.	Très influent	Un peu influent	Neutre	Pas très influent	Pas du tout influent	N/A
Je pense que je suis sujette à une infection de VPH.	Très influent	Un peu influent	Neutre	Pas très influent	Pas du tout influent	N/A
Il y a des antécédents d'anomalies du col de l'utérus dans ma famille.	Très influent	Un peu influent	Neutre	Pas très influent	Pas du tout influent	N/A
Je connais quelqu'un qui a/avait le cancer du col de l'utérus.	Très influent	Un peu influent	Neutre	Pas très influent	Pas du tout influent	N/A
Mes parents me l'ont recommandé.	Très influent	Un peu influent	Neutre	Pas très influent	Pas du tout influent	N/A
Mon (mes) amie(s) me l'a(ont) recommandé.	Très influent	Un peu influent	Neutre	Pas très influent	Pas du tout influent	N/A
Le vaccin était couvert par mon assurance maladie.	Très influent	Un peu influent	Neutre	Pas très influent	Pas du tout influent	N/A
Le vaccin m'était offert à l'école (primaire, secondaire).	Très influent	Un peu influent	Neutre	Pas très influent	Pas du tout influent	N/A
Le coûte du vaccin.	Très influent	Un peu influent	Neutre	Pas très influent	Pas du tout influent	N/A
La plupart des filles/femmes mon âge recevait le vaccin.	Très influent	Un peu influent	Neutre	Pas très influent	Pas du tout influent	N/A
Le vaccin pourrait me protéger contre une infection par le VPH	Très influent	Un peu influent	Neutre	Pas très influent	Pas du tout influent	N/A
Le vaccin pourrait me protéger contre le développement des verrues génitales.	Très influent	Un peu influent	Neutre	Pas très influent	Pas du tout influent	N/A
Le vaccin pourrait me protéger contre le développement du cancer du col de l'utérus.	Très influent	Un peu influent	Neutre	Pas très influent	Pas du tout influent	N/A

30) Si votre assurance maladie ne le couvait pas, quel serait le coût maximal que vous pourriez à payer pour la série de vaccins contre le VPH?

- ☐ 0$
- ☐ Plus que 0$ mais moins ou égalé à 100$
- ☐ 200$
- ☐ 300$
- ☐ 400$
- ☐ Plus que ou égalé à 500$
- ☐ Je ne recevrai pas le vaccin.

Section 3 : L'acceptabilité du vaccin et la pratique des rapports sexuels sans risque
Dans cette section, nous voulons savoir comment ce vaccin pourra changer votre future santé et vos rapports sexuels sans risque.

31) Recevoir le vaccin contre le VPH me porte/ me portera moins à recevoir des tests de Papanicolaou.

- ☐ Fermement en accord
- ☐ En accord
- ☐ Neutre
- ☐ En désaccord
- ☐ Fermement en désaccord

32) En supposant que vous avez reçu ou que vous étiez prête à recevoir le vaccin contre le VPH, SVP indiquez à quel point vous êtes en accord ou en désaccord avec les affirmations suivantes, au sujet des rapports sexuels sans risque. Complétez la déclaration :
"Après avoir reçu le vaccine contre le VPH ________________"

Je pense que l'utilisation d'un condom durant les relations sexuelles est moins nécessaire.	Fermement en accord	En accord	Neutre	En désaccord	Fermement en désaccord
Je pense que c'est tout aussi important d'avoir le moins de partenaires sexuels possible.	Fermement en accord	En accord	Neutre	En désaccord	Fermement en désaccord
Je pense que c'est moins important de parler avec mes partenaires sexuels au sujet des rapports sexuels sans risque.	Fermement en accord	En accord	Neutre	En désaccord	Fermement en désaccord
Je pense que c'est tout aussi important d'utiliser un condom à chaque rapport sexuel.	Fermement en accord	En accord	Neutre	En désaccord	Fermement en désaccord
Je m'inquiéterai moins des rapports sexuels non-protégés.	Fermement en accord	En accord	Neutre	En désaccord	Fermement en désaccord

33) Est-ce que recevoir le vaccin contre le VPH a affecté/affectera combien de fois vous utiliserez une sorte de contrôle des naissances ou de la contraception durant les rapports sexuels, comparé à avant de recevoir le vaccin?

- ☐ Oui, je l'utiliserai plus souvent.
- ☐ Oui, je l'utiliserai moins souvent.
- ☐ Non, je ne changerai pas la fréquence avec laquelle j'utilise le contrôle des naissances/contraception.
- ☐ Je ne sais pas.
- ☐ N/A

Section 4 : Connaissance du VPH et du cancer du col de l'utérus
Il y a 2 vaccins différents contre le VPH qui sont disponible pour les femmes. Ces deux vaccins, Gardasil® et Cervarix®, fournissent une protection contre les souches les plus communes du VPH causant le cancer du col de l'utérus. De plus, Gardasil® protège contre deux autres souches du VPH qui causent seulement des verrues génitales.

Dans cette section, nous voulons savoir si vous êtes au courant de certains faits concernant VPH et du cancer du col de l'utérus.

34) Indiquez si vous pensez que les déclarations suivantes sont vraies ou fausses.

Le VPH est principalement transmit de manière sexuelle.	Vrai	Faux
Le VPH est la plus commune des IST (infections sexuellement transmissibles).	Vrai	Faux
Le VPH affecte seulement les femmes.	Vrai	Faux
La plupart des personnes infectée avec le VPH ne démontre pas de signes ou de symptômes d'une infection .	Vrai	Faux
La vaccination contre le VPH est seulement efficace si vous n'êtes pas sexuellement active.	Vrai	Faux
Le taux le plus élevé des infections au VPH est chez les adultes de <25 ans.	Vrai	Faux
Une infection avec le VPH peut causer le cancer du col de l'utérus.	Vrai	Faux
3 doses du vaccin contre le VPH sont requises pour vous assurer de la protection complète.	Vrai	Faux
Au moins 1 femme sur 2 est diagnostiquée avec le VPH durant sa vie.	Vrai	Faux
La vaccination contre le VPH vous protège contre toutes les infections reliées au VPH.	Vrai	Faux
Le signe le plus commun d'une infection de VPH est la présence de verrues génitales.	Vrai	Faux
La vaccination contre le VPH est efficace seulement pour les femmes.	Vrai	Faux
L'infection avec le VPH est associée avec plusieurs types de cancers.	Vrai	Faux
2 souches du VPH expliquent 70% des cancers du col de l'utérus.	Vrai	Faux
Le VPH est la seule cause établi du cancer du col de l'utérus.	Vrai	Faux
Les tests de Pap ne sont pas nécessaires si les femmes sont vaccinées contre le VPH.	Vrai	Faux

35) Avant aujourd'hui, étiez-vous au courant qu'il avait des vaccins disponibles qui pouvaient prévenir le cancer du col de l'utérus et d'autres infections associées avec le VPH?
- ☐ Oui
- ☐ Non

36) Avant aujourd'hui, étiez-vous au courant qu'il avait 2 vaccins différents disponibles contre le VPH?
- ☐ Oui
- ☐ Non

Section 5 : Données démographiques
Dans cette dernière section, nous voulons savoir un peu plus sur vous.

37) Dans quelle faculté étudiez-vous?
- ☐ Arts
- ☐ Sciences
- ☐ Génie
- ☐ Sciences de la santé
- ☐ Autre (SVP spécifiez : _____________________)

38) Quel âge avez-vous?
- ☐ Moins que 18 ans
- ☐ 18 ans
- ☐ 19 ans
- ☐ 20 ans
- ☐ 21 ans
- ☐ 22 ans
- ☐ 23 ans
- ☐ 24 ans
- ☐ 25 ans
- ☐ Plus que 25 ans

39) Vous pouvez être un membre d'un ou plusieurs groupes ethniques ou culturels de la liste suivante. Quels sont vos origines culturelles ou ethniques?
- ☐ Blanc
- ☐ Asiatique-sud (ex. est-Indienne, Pakistanaise, Sri Lankan etc.)
- ☐ Chinoise
- ☐ Noire
- ☐ Philippine
- ☐ Latino-Américaine
- ☐ Arabe
- ☐ Asiatique sud-est (ex : Vietnamienne, Cambodgienne, Malaysienne, Laotienne etc.)
- ☐ Asiatique-ouest (ex : Iranienne, Afghane etc.)
- ☐ Coréenne
- ☐ Japonaise
- ☐ Aborigène (inclut Première Nation/Inuit/Métis)
- ☐ Autre (SVP spécifiez : _____________________)

40) Avant de commencer vos études à l'Université d'Ottawa, ou viviez-vous?
- ☐ Alberta
- ☐ Colombie-Britannique
- ☐ Manitoba
- ☐ Nouveau Brunswick
- ☐ Terre Neuve et Labrador
- ☐ Nouvelle-Écosse
- ☐ Ontario

☐ Ile-du-Prince Édouard
☐ Québec
☐ Saskatchewan
☐ Territoires du Nord-Ouest
☐ Nunavut
☐ Yukon
☐ Autre pays (SVP spécifiez :______________________)

41) SVP indiquez la réponse qui correspond le mieux à votre assurance maladie, autre que celle fourni par la province (OHIP, RAMQ).
☐ Je suis couverte par le régime d'assurance maladie de mon(mes) parent(s) ou mon tuteur.
☐ Je suis couverte par le régime d'assurance maladie de l'université.
☐ J'ai mon propre régime d'assurance maladie privée.
☐ Je suis couverte par le régime d'assurance maladie de mon époux.
☐ Je n'ai pas de régime d'assurance maladie.

42) Avez-vous déjà eu des rapports sexuels (oraux, vaginaux ou anaux) avec un homme?
☐ Oui
☐ Non

43) Avez-vous déjà eu un test de Papanicolaou?
☐ Oui, plus d'une fois.
☐ Oui, une fois.
☐ Non
☐ Je ne sais pas.

44) Avez-vous déjà eu un test de Papanicolaou anormal?
☐ Oui
☐ Non
☐ Je ne sais pas.

45) Êtes-vous à jour dans vos vaccins réguliers (ex. avez-vous reçu vos vaccins de rappel)?
☐ Oui
☐ Non
☐ Je ne sais pas.

46) Est-ce que vous utilisez une sorte de contrôle des naissances/contraception?
☐ Oui
☐ Non

47) Est-ce qu'un professionnel de la santé vous avez déjà dit que vous aviez une infection par le VPH?
☐ Oui
☐ Non

48) Est-ce qu'un professionnel de la santé vous avez déjà dit que vous aviez une IST (infection sexuellement transmissible) autre que le VPH?
☐ Oui

- ☐ Non
- ☐ Je ne sais pas.

49) Êtes-vous nées au Canada?

- ☐ Oui
- ☐ Non
